# Fibroblasts Colonizing Nerve Conduits Express High Levels of Soluble Neuregulin1, a Factor Promoting Schwann Cell Dedifferentiation

**DOI:** 10.3390/cells9061366

**Published:** 2020-06-01

**Authors:** Benedetta E. Fornasari, Marwa El Soury, Giulia Nato, Alessia Fucini, Giacomo Carta, Giulia Ronchi, Alessandro Crosio, Isabelle Perroteau, Stefano Geuna, Stefania Raimondo, Giovanna Gambarotta

**Affiliations:** 1 Department of Clinical and Biological Sciences (DSCB), University of Torino, 10043 Orbassano (Torino), Italy; benedettaelena.fornasari@unito.it (B.E.F.); marwa.elsoury@unito.it (M.E.S.); alessia.fucini@edu.unito.it (A.F.); giacomo.carta@unito.it (G.C.); giulia.ronchi@unito.it (G.R.); alessandro.crosio@unito.it (A.C.); isabelle.perroteau@unito.it (I.P.); stefano.geuna@unito.it (S.G.); stefania.raimondo@unito.it (S.R.); 2 Neuroscience Institute Cavalieri Ottolenghi (NICO), University of Torino, 10043 Orbassano (Torino), Italy; giulia.nato@unito.it; 3 Department of Life Sciences and Systems Biology (DBIOS), University of Torino, 10123 Torino, Italy; 4 Hand Surgery and Reconstructive Microsurgery, Gaetano Pini—CTO Hospital, 20122 Milano, Italy

**Keywords:** Neuregulin 1, nerve fibroblasts, nerve regeneration, Schwann cells, nerve guide, chitosan, peripheral nerve, nerve repair, nerve injury

## Abstract

Conduits for the repair of peripheral nerve gaps are a good alternative to autografts as they provide a protected environment and a physical guide for axonal re-growth. Conduits require colonization by cells involved in nerve regeneration (Schwann cells, fibroblasts, endothelial cells, macrophages) while in the autograft many cells are resident and just need to be activated. Since it is known that soluble Neuregulin1 (sNRG1) is released after injury and plays an important role activating Schwann cell dedifferentiation, its expression level was investigated in early regeneration steps (7, 14, 28 days) inside a 10 mm chitosan conduit used to repair median nerve gaps in Wistar rats. In vivo data show that sNRG1, mainly the isoform α, is highly expressed in the conduit, together with a fibroblast marker, while Schwann cell markers, including NRG1 receptors, were not. Primary culture analysis shows that nerve fibroblasts, unlike Schwann cells, express high NRG1α levels, while both express NRG1β. These data suggest that sNRG1 might be mainly expressed by fibroblasts colonizing nerve conduit before Schwann cells. Immunohistochemistry analysis confirmed NRG1 and fibroblast marker co-localization. These results suggest that fibroblasts, releasing sNRG1, might promote Schwann cell dedifferentiation to a “repair” phenotype, contributing to peripheral nerve regeneration.

## 1. Introduction 

Traumatic injury to peripheral nerve represents a relevant clinical problem that can lead to permanent disability and functional impairment; indeed, it has been estimated that ~3% of all trauma involve peripheral nerves [1]. Unlike the central nervous system, peripheral nerves regenerate spontaneously after a crush (axonotmesis) or a clear cut (neurotmesis), when proximal and distal stumps remain in close contact, facilitating the formation of a bridge and axonal re-growth [2,3]. Nevertheless, after a clear cut, a microsurgical intervention is always recommended to guide the re-growing axons from the proximal to the distal stump and then to their target. If it is possible to connect the two stumps without tension, a direct suture is suggested; if, following nerve tissue loss, a direct suture would cause a too high tension, it is necessary to insert a scaffold to bridge the two stumps without tension [4]. The length of the gap which needs bridging with a scaffold varies according to the characteristics of different nerves and their anatomical district [5]. 

Despite the progresses reached in nerve repair techniques during recent decades, peripheral nerve gap repair remains a significant clinical challenge. For severe injuries, the gold standard technique is the autograft, i.e., transplantation of an autologous nerve, but it presents some side effects, such as a secondary surgery and sensitivity loss [6]. A good alternative is the tubulization, where a tubular conduit is used to connect the gap between the nerve stumps. Different tubular conduits have been developed and several are commercially available, as recently reviewed [7,8]. Conduit repair efficiency is similar to the autograft for short distance repair, while for long distances the autograft remains the most effective technique [9,10]. Indeed, autograft, being a transplanted nerve, yet contains most of the players involved in Wallerian degeneration and nerve regeneration, while conduits require colonization by Schwann cells, fibroblasts, endothelial cells, and so on. A promising biomaterial widely used for peripheral nerve regeneration and recently approved for clinical use is chitosan, a polysaccharide of natural origins [10,11,12]. It is a highly versatile biomaterial, indeed it has been tested in several forms, such as hydrogels, films, or conduits used to repair nerve gap [13].

After a nerve cut, a bridge spontaneously forms between the proximal and the distal stumps; the analysis of the bridge showed that, during nerve regeneration, important roles are played by macrophages, endothelial cells, Schwann cells, and fibroblasts [14]. Initially, the bridge, composed by inflammatory cells and extracellular matrix released by fibroblasts, is poorly vascularized and becomes hypoxic: Macrophages, sensing hypoxia, release vascular-endothelial growth factor (VEGF) which attracts endothelial cells promoting angiogenesis; blood vessels provide a track for Schwann cell migration [14]. In the degenerating distal stump, Schwann cells, releasing soluble NRG1, might promote their de-differentiation from a myelinating to a “repair” phenotype, playing a key role in myelin debris clearing, macrophage attraction, neuron survival, and axonal re-growth [15]. Indeed, soluble NRG1 has been shown to play an important role for nerve regeneration [16], being highly, and transiently, over-expressed immediately after injury [17,18] and negatively affecting nerve regeneration when it is missing [18]. Different NRG1 isoforms obtained by alternative splicing were described [19]: They are mainly classified as transmembrane (expressed by axons and strongly involved in the regulation of myelination) or soluble (released by Schwann cells immediately after nerve injury), and as type α or type β, according to the C-terminus of EGF-like domain, the domain which is involved in the interaction with NRG1 receptors, namely ErbB3 and ErbB4 [20,21]. In Schwann cells, NRG1 signal transduction is mediated by the heterodimeric receptor ErbB2-ErbB3 [22].

While many studies have contributed to our knowledge about how peripheral nerves regenerate in case of mild injuries, such as crush (axonotmesis) or clear-cut transections (neurotmesis) followed by end-to-end repair, much less is understood about nerve regeneration after a severe injury with loss of nerve tissue. In particular, although tubulization is often and successfully used as a good alternative to autograft for short nerve gap repair [7,10,12,23,24,25,26,27], little is known about what happens during regeneration inside a conduit used to repair this kind of injury. A description of the different stages occurring inside a hollow conduit during nerve regeneration was carried out several years ago by means of electron microscopy analysis [28]: (1) The conduit is initially filled with plasma exudate which contains extracellular matrix molecules and neurotrophic factors; (2) fibrin filaments are formed; (3) fibroblasts and dedifferentiated Schwann cells colonize the gap, proliferate and align along the conduit forming the Bands of Bϋngner; (4) axons grow and reach their distal targets exploiting the biological cues supplied by Schwann cells; and (5) Schwann cells differentiate to a myelinating phenotype and myelinate axons [29]. 

Our aim was to further characterize the stages occurring inside a conduit using different techniques to identify the cells and the factors involved in the nerve regeneration, focusing our attention on the NRG1/ErbB system, with the idea that understanding what happens inside a nerve conduit might contribute to develop new strategies to further promote nerve repair.

## 2. Materials and Methods

### 2.1. Surgical Procedure 

Eighteen adult female Wistar rats (ENVIGO, Milan, Italy), weighing about 200–250 g, were used. The total number includes animals used for uninjured control nerve withdrawal. Animals were kept under standardized and controlled conditions (12 h of light and 12 h of dark and free access to water and food). Surgeries were performed under general anesthesia through intraperitoneal injection of tiletamine HCl and zolazepam HCl (Zoletil Virbac, Milan, Italy) + xilazine (Rompun, Bayer, Leverkusen, Germany) (40 mg kg^−1^ + 5 mg kg^−1^). 

The median nerve was exposed and transected to establish a defect (8 mm of nerve was removed). Then, two experimental groups were obtained: the chitosan conduit group and the autograft group as previously described [30] (Figure S1). 

1-for the chitosan conduit group a hollow 10 mm long chitosan tube was used to bridge the 8 mm nerve defect by inserting 1 mm of each nerve end inside the conduit (8 mm gap was chosen considering it). The nerve conduit was sutured with one 9/0 epineurial stitch (silk; Péters Surgicals, Bobigny, France) at each end. Chitosan-based conduits (Reaxon^®^ Nerve Guides) were supplied by Medovent GmbH (Mainz, Germany). 

2-for the autograft group, the transected median nerve segment was reversed (distal-proximal) and sutured to the nerve ends of the same animal with three 9/0 epineurial stitch at each end. Regenerated nerves were withdrawn 7, 14, and 28 days after the repair for biomolecular analysis (*n* = 3–4 for each group) and 7 days after the repair for morphological analysis; then, animals were sacrificed by anesthetic overdose (>100 mg kg^−1^ Zoletil and 30 mg kg^−1^ Rompun).

Control nerves were healthy median nerves obtained from 4 uninjured animals.

### 2.2. Ethics Approval and Consent to Participate

Animal study followed the recommendations of the Council Directive of the European Communities (2010/63/EU), the Italian Law for Care and Use of Experimental Animals (DL26/14), and are in agreement with the National Institutes of Health guidelines (NIH Publication No. 85-23, revised 1996). All animal experiments were carried out at the animal facility of Neuroscience Institute Cavalieri Ottolenghi (NICO) (Ministerial authorization DM 182 2010-A 3-11-2010). The current experimental study was reviewed and approved by the Ethic Experimental Committee of the University of Torino (Italian Ministry of Health approved project number: 864/2016/PR, 14-09-2016). 

### 2.3. Schwann Cell Primary Culture 

To obtain adult primary Schwann cell culture, 4 rat sciatic nerves were isolated for each biological replicate (*n* = 3). The *epineurium* was removed, nerves were cut into small pieces about 1 mm long, then were evenly distributed in a 3 cm diameter Petri dish and were incubated for 24 h in dissociation medium Dulbecco Modified Eagle Medium (DMEM, Gibco, Thermo Fisher Scientific, Waltham, MA, USA) containing 1 g/L glucose, 10% heat-inactivated fetal bovine serum (FBS; Invitrogen, Thermo Fisher Scientific), 100 units/mL penicillin, 0.1 mg/mL streptomycin, 10 µM Forskolin, 63 ng/mL recombinant NRG1β1 (#396-HB, R&D Systems, Minneapolis, MN, USA), 0.625 mg/mL collagenase IV, 0.5 mg/mL dispase II at 37 °C in a 5% CO_2_ atmosphere saturated with H_2_O.

After 24 h, mechanical dissociation was performed and the medium containing the dissociated nerves was collected in a tube, then the suspension was filtered through a cell strainer with 70 µm pores (Sartorius Stedim Biotech GmbH, Göttingen, Germany) and transferred into a new tube. Cells were centrifuged at 100 rcf for 5 min. The pellet obtained was resuspended in DMEM D-valine medium (Cell Culture Technologies, Gravesano, Switzerland) containing D-valine, 4.5 g/L glucose, 2 mM glutamine, 10% FBS, 100 units/mL penicillin, 0.1 mg/mL streptomycin, 10 µM Forskolin, and 63 ng/mL NRG1β1. Cells were grown in a cell culture dish pre-treated with poly-L-lysine (PLL) to allow Schwann cell adhesion, at 37 °C in a 5% CO_2_ atmosphere saturated with H_2_O. Medium was replaced every two days. Cells (passage 1) were allowed to proliferate until confluence, then split and allowed to proliferate until confluence in a 6 cm diameter Petri dish (passage 2) for the subsequent extraction with TRIzol Reagent (Invitrogen, Thermo Fisher Scientific) to obtain RNA and protein, as described below. 

DMEM D-valine medium was used to obtain Schwann cells, as the essential amino acid D-valine in this media can be exclusively metabolized by Schwann cells and not by fibroblasts, owing to the expression of the D-amino acid oxidase (DAAO) enzyme in Schwann cells. Since fibroblasts are not able to metabolize this isoform, they die after a few days in culture, due to the lack of an essential amino acid [31]. Unless specified, all reagents were purchased from Sigma-Aldrich, Merck, Darmstadt, Germany.

### 2.4. Nerve Fibroblast Primary Culture 

To obtain adult primary nerve fibroblasts 2 rat sciatic nerves were isolated for each biological replicate (*n* = 3). The protocol is similar to that used for Schwann cell isolation, except for: (i) The epineurium was not removed from sciatic nerves, (ii) the culture medium DMEM (Sigma-Aldrich, Merck) contained L-valine, 4.5 g/L glucose, 10% FBS, 2 mM L-glutamine and 100 units/mL penicillin, 0.1 mg/mL streptomycin, and (iii) fibroblasts were cultured without any coating. Medium was replaced every two to three days. At least three passages were carried out to reduce the number of contaminating Schwann cells and to increase the purity of the primary culture. The purity of the culture was assessed by immunohistochemistry (data not shown). After reaching confluence, RNA and proteins were extracted for subsequent expression analysis, as described below. 

Sciatic nerves to obtain Schwann cell and fibroblast primary cultures were withdrawn from nine uninjured animals, four of which were the animals used for control group median nerve withdrawal.

### 2.5. RNA Isolation, cDNA Preparation, and Quantitative Real-Time PCR

Total RNA extraction and retrotranscription, and quantitative real-time PCR (qRT-PCR), were performed as previously described [17], using 0.75 µg /sample for retrotranscription. Technical and biological triplicates were performed for in vitro sample analysis, while for in vivo sample analysis, biological replicates were 4. qRT-PCR data were analyzed using the “Livak 2^−ΔΔCt^ method” for the relative quantification [32]. Briefly, the threshold cycle (C_t_) values of both the calibrator and the samples of interest were normalized to housekeeping genes to obtain ΔC_t_, then ΔC_t_ were normalized to the calibrator to obtain ΔΔC_t_ and, following, the relative quantification (2^−ΔΔCt^) representing the expression level of each sample relatively to the calibrator sample. As calibrator for the relative quantification the average of uninjured nerve was used. The geometric average of the two endogenous housekeeping genes ANKRD27 (Ankyrin repeat domain 27) and RICTOR (RPTOR Independent Companion Of MTOR, Complex 2) was used for data normalization [33]. 

Primer sequences for ErbB1, ErbB2, ErbB3, soluble NRG1, ANKRD27, RICTOR [17], ErbB4, NRG1α, NRG1β [30], S100, and p75 [34] were previously published. A new primer pair for Thy1 (accession number #NM012673, amplicon length 127 bp) was prepared: Thy1 forward: 5′-CTCCTGCTTTCAGTCTTGCAGATGTC-3′; Thy1 reverse: 5′-CATGCTGGATGGGCAAGTTGGTG-3′. 

### 2.6. Protein Extraction and Western Blot

After RNA extraction, total proteins were extracted by the TRIzol Reagent (Invitrogen, Thermo Fisher Scientific) according to manufacturer′s instructions, then, protein pellet was dissolved in boiling LaemLi buffer (2.5% sodium dodecyl sulphate, 0.125 M Tris-HCl, pH 6.8). The Bicinchoninic Acid assay kit (Sigma-Aldrich, Merck) was used to determine protein concentration and equal amounts of proteins (50 μg) were loaded into each lane. Proteins were resolved by 8% SDS-PAGE. Western blot analysis was carried out as previously described [35]. The list of primary and secondary antibodies is reported in Table S1. Secondary antibodies are horseradish peroxidase-linked.

### 2.7. Immunohistochemistry 

Seven days after the surgery, median nerves from each experimental group were withdrawn for the following immunohistochemistry (IHC) analysis. Before fixation chitosan conduits were removed with high precision in order to avoid loss of the tissue grown inside. Regenerated nerve specimens were fixed in 4% paraformaldehyde in PBS (phosphate buffered saline) (Sigma-Aldrich, Merck) for 2–3 h and stored in PBS at 4 °C. For the Cryo-embedding procedure, samples were cryo-protected with passages in solutions with increasing concentration of sucrose (Sigma-Aldrich, Merck, 7.5% for 1 h, 15% for 1 h, 30% overnight) in 0.1 M PBS. Next, samples were maintained in a solution of 30% sucrose and optimal cutting temperature medium (OCT, Electron Microscopy Sciences, Hatfield, PA, USA) in a ratio 1:1, for 30 min and then embedded in 100% OCT; samples were stored at −80 °C. Nine micrometer-thick cross sections of the nerve were obtained starting from the middle of the autograft or the middle of the new tissue grown inside the chitosan conduit.

For IHC, samples on slices were rinsed with PBS and then permeabilized and saturated in 0.01% Triton X-100, 10% normal donkey serum (NDS, Jackson ImmunoResearch, Philadelphia, PA, USA) in PBS for 1 h at room temperature in humid chamber. Samples were incubated with primary antibodies (Table S1, double staining were performed in single step, combined as follows: CD34 + S100β, CD34 + NRG1, CD34 + RECA1) at 4 °C overnight in PBS containing 1% NDS, in humid chamber. Then, samples were rinsed three times with PBS and incubated 1h at room temperature in humid chamber with secondary antibodies (Table S1). After three washes in PBS, sections were covered with a mounting solution (Fluoromount, Sigma-Aldrich, Merck) and a glass coverslip was used to cover the section before observation. After drying for at least 3 days, samples were photographed using Leica (Wetzlar, Germany) TCS SP5 confocal microscope. 

For specificity assessment, all these antibodies were previously checked by Western blotting, which showed a single band of staining (data not shown). These antibodies have been used for similar purposes in the literature. Anti-S100β was used to label Schwann cells [36], anti-CD34 was used to specifically label nerve fibroblasts [37], even if it known to recognize also endothelial cells. As shown below in the immunohistochemistry panel 4C, no cells co-express S100β and CD34, (indicating that these antibodies do not cross react). To specifically identify fibroblasts, anti-CD34 was coupled with anti-RECA1, a cell surface antigen which is expressed by endothelial cells [38,39].

To assess the expression of NGR1 in fibroblasts, slices were stained with anti-CD34 and anti-NRG1, a polyclonal antibody that recognizes the C-terminal of many different NRG1 isoforms (all isoforms with “type a” C terminus, which is common to many soluble isoforms) [19], used by several laboratories and acknowledged by the scientific community as a suitable antibody to assess NRG1 expression [18,40].

### 2.8. Statistical Methods

For in vivo experiments a priori power analysis performed with G*Power (v. 3.1.9.4; Heinrich-Heine-Universität Düsseldorf, Germany) [41] showed that a sample size of 12 samples (*n* = 4 for each group) for each time point was determined respecting an effect size of *d* = 0.6, with a power of 0.95 and α of 0.05 defining an actual power of 0.98 to limit the number of sacrificed animals. 

Statistical analyses were performed with IBM SPSS Statistics 25 (IBM, Armonk, NY, USA) software. Data were expressed as mean ± standard error of the mean (SEM). All data were tested for normal distribution in order to select the appropriate statistical test (Levene and Mauchly tests).

Analysis of variance (ANOVA) for repeated measures with Bonferroni’s correction was adopted to detect the effect of time, experimental groups, and their interaction and to highlight the overall significant differences among autograft group, chitosan group and uninjured control nerves and at each time point analyzed. 

The two-tailed Student’s *t*-test for normally distributed data with comparable variances was used to highlight statistical differences between the two groups (Schwann cells and fibroblasts), while for data not showing normal distribution the nonparametric Mann–Whitney U-test was used. 

The effect size of each factor was computed as partial eta-squared (η_p_^2^): a small effect was considered for a value of 0.010, a medium effect for value of 0.059 and a large effect for value of 0.138 [42].

## 3. Results

### 3.1. Soluble NRG1 Is Strongly Expressed in the Conduit after Nerve Repair, While ErbB2 and ErbB3 Are Missing

**Figure 1:**
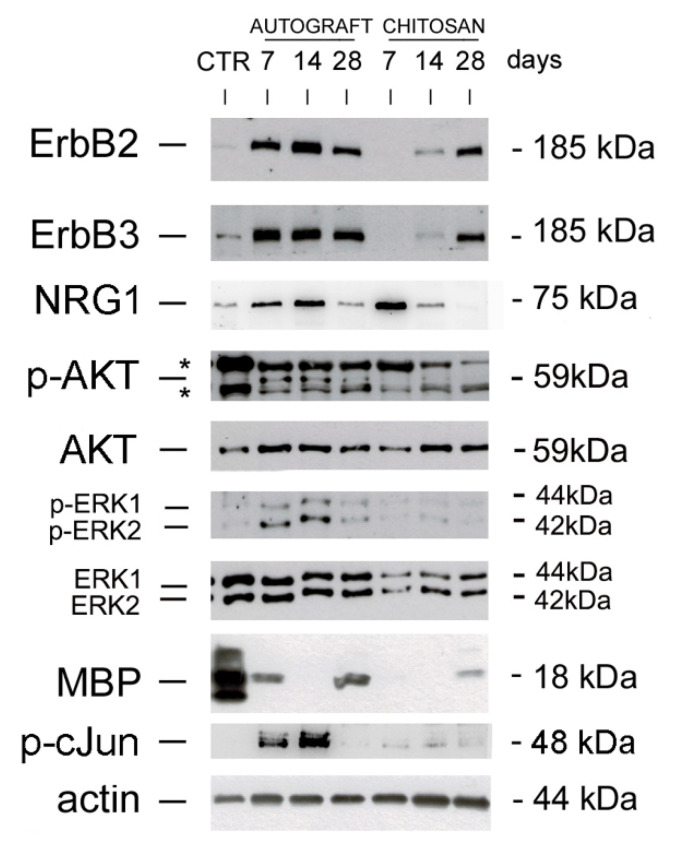
NRG1 and ErbB expression in autograft and chitosan groups. Western blot analysis of proteins extracted from healthy control nerves (CTR) and regenerating nerves belonging to autograft or chitosan groups withdrawn 7, 14, and 28 days after the repair and probed with antibodies for ErbB2, ErbB3, phospho-AKT, total-AKT, phospho-ERK, total-ERK, MBP, phospho-cJun; actin was used as a loading control. For NRG1, an antibody recognizing the C terminus fragment was used. Unspecific bands were identified with asterisks (*). Molecular weight (kDa) are shown on the right.

The expression of proteins belonging to the NRG1/ErbB system, which is strongly involved in peripheral nerve regeneration, was investigated in the new tissue grown inside the chitosan conduit (“chitosan” group) and compared with the grafted autologous nerve (“autograft” group), representing the surgery “gold-standard”, or with healthy median nerve, used as control group, representing the starting point and the aimed final outcome (Figure 1).

Our data show that ErbB2 and ErbB3 receptors were strongly up-regulated in the autologous nerve graft at all the time points analyzed (7, 14, and 28 days after the repair), while in the chitosan conduit ErbB2 and ErbB3 were not detectable 7 days after repair; they started to be detectable one week later, and were strongly expressed 28 days after the repair.

To detect soluble NRG1, an antibody which recognizes a 75 kDa band corresponding to the C terminal fragment of NRG1 was used [17,18]. NRG1 is strongly expressed 7 and 14 days after the repair in both groups, with a higher expression in the chitosan group 7 days after the repair. NRG1 expression strongly decreases 28 days after the repair in both experimental groups. 

### 3.2. The PI3K/AKT and ERK/MAPK Pathways Are Activated in the Autograft, Not in the Conduit

AKT, ERK and cJun are strongly phosphorylated 7 and 14 days after repair with the autograft technique, while after 28 days their phosphorylation strongly decreases (AKT, ERK) or it is completely switched off (cJun). Inside the conduit, AKT phosphorylation is barely detectable 14 days after the repair, while ERK1/2 and cJun phosphorylation are not detectable; moreover, in the chitosan samples the total amount of ERK is lower than in the autograft. 

Myelin basic protein (MBP) is strongly expressed in the uninjured median nerve; in the autograft group it is still present 7 days after injury (during Wallerian degeneration), disappears 14 days after repair (during axon regrowth) and it is present again 28 days after the repair (during axon remyelination). Within the chitosan conduit, where Wallerian degeneration does not occur, MBP starts to be present 28 days after the repair, during axon remyelination.

### 3.3. Fibroblast Markers Are Highly Expressed in the Chitosan Conduit 

**Figure 2:**
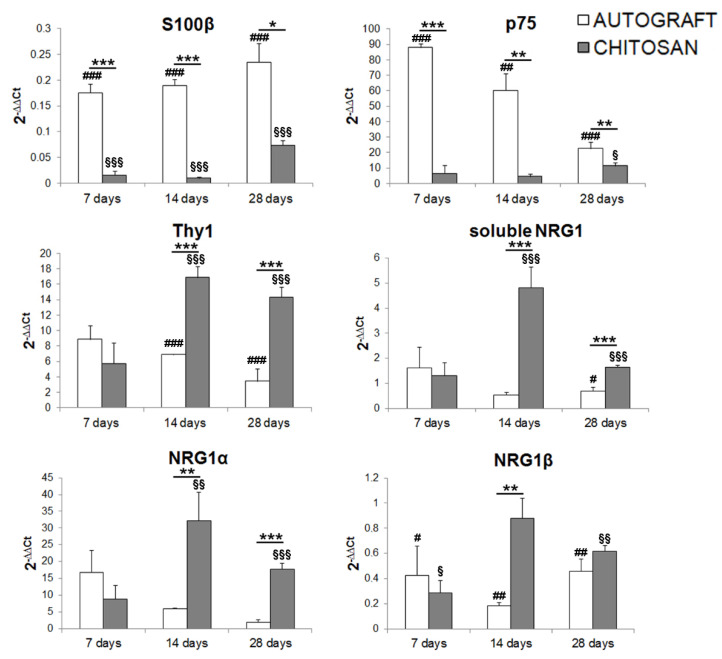
Quantitative expression analysis of Schwann cell and nerve fibroblast markers. Relative quantification (2^−ΔΔCt^) of S100β and p75 (Schwann cell markers), Thy1 (fibroblast marker), soluble NRG1, NRG1α, and NRG1β was evaluated by qRT-PCR. The geometric average of the housekeeping genes ANKRD27 and RICTOR was used to normalize data. Values in the graphics are expressed as mean + SEM (*n* = 3–4 for each group). ANOVA for repeated measures with Bonferroni’s correction was adopted for statistical analysis; * denotes the significant differences between autograft and chitosan groups at each time point analyzed (*p ≤ 0.05, **p ≤ 0.01, and ***p ≤ 0.001); # denotes the significant differences between autograft group and uninjured control nerves at each time point analyzed (^#^*p* ≤ 0.05, ^##^*p* ≤ 0.01, and ^###^*p* ≤ 0.001); § denotes the significant differences between chitosan group and uninjured control nerves at each time point analyzed (^§^*p* ≤ 0.05, ^§§^*p* ≤ 0.01, and ^§§§^*p* ≤ 0.001). All data are calibrated to uninjured nerves (whose expression, not shown, is = 1).

Western blot data analysis showed that in the chitosan conduit soluble NRG1 (usually highly expressed by Schwann cells after injury) was strongly expressed 7 days after nerve repair, while the NRG1 co-receptors ErbB2-ErbB3 were not. To identify the cells responsible for NRG1 release, we analyzed the expression of two Schwann cell (S100β, p75) and one fibroblast (Thy1) markers within the chitosan conduit (Figure 2 and Table 1 for statistical analysis). 

S100β, a Schwann cell marker highly expressed by differentiated Schwann cells, is significantly down-regulated after injury in all samples. Our data show that at all the time points analyzed S100β is significantly less expressed in conduit samples than in the nerve repaired through autologous graft. The effect size between all groups is significantly large for all time points analyzed (see Table 1). Multiple comparison analysis for S100β revealed significant differences between autograft and chitosan groups (*p* = 0.0056, mean difference = 0.16, CI 95% = 0.06–0.26). The within subject test showed no significant effect for time (*p* = 0.065) and time x group factors (*p* = 0.29). 

p75 is a Schwann cell marker highly expressed by dedifferentiated Schwann cells, known to be strongly up-regulated after nerve injury; accordingly, when compared with uninjured nerves, p75 expression is significantly higher in all autograft samples, while in the chitosan group it is significantly more highly expressed only 28 days after the repair (see Table 1). Data show that at all the time points analyzed p75 is significantly less expressed in the conduit group than in the nerve repaired through autologous graft. The effect size between experimental groups is large for all time points analyzed (see Table 1). Multiple comparison analysis for p75 revealed significant differences between the autograft and the chitosan groups (*p* = 0.000016, mean difference = 47.15, CI 95% = 36.93–57.37) and between the autograft and the control groups (*p* = 0.000007, mean difference = 54.43, CI 95% = 44.21–64.65). The within subject test revealed a significant effect for time (*p* = 0.002) and time x group (*p* = 0.0004) factors.

The nerve fibroblast marker Thy1 is significantly more highly expressed in the chitosan group, 14 and 28 days after the repair with a large effect size (see Table 1), in comparison with the autograft group and with uninjured nerves. Multiple comparison analysis for Thy1 revealed significant differences between the autograft and the chitosan groups (*p* = 0.004, mean difference = −6.5, CI 95% = (−10.36)–(−2.62)), between the autograft and the control groups (*p* = 0.012, mean difference = 5.3, CI 95% = 1.49–9.23) and between the chitosan and the control groups (*p* = 0.00017, mean difference = 11.86, CI 95% = 7.98–15.72). The within subject test revealed a significant effect for time x group factors (*p* = 0.01), not for time alone (*p* = 0.15).

Soluble NRG1 is significantly higher in the chitosan group compared with the autograft group and with uninjured nerves 14 and 28 days after repair with large effect size (see Table 1). Multiple comparison analysis for soluble NRG1 revealed significant differences between chitosan and uninjured nerve groups (*p* = 0.0002, mean difference = 1.88, CI 95% = 1.18–2.58) and between chitosan and autograft groups (*p* = 0.0002, mean difference = 1.96, CI 95% = 1.21–2.70). The within subject test revealed a significant effect for time (*p* = 0.006) and time x group (*p* = 0.000) factors. 

Soluble NRG1 isoforms (which are the most represented isoforms, at the mRNA level, in the peripheral nerve) include both α and β isoforms, whose regulation and activity can be different; therefore, we analyzed, separately, the expression of both isoforms. 

NRG1α is more highly expressed in the chitosan group, in comparison with the autograft group and with uninjured nerves, 14 and 28 days after the repair with large effect size (see Table 1). Multiple comparison analysis for soluble NRG1α revealed significant differences between the chitosan and the uninjured nerve groups (*p* = 0.002, mean difference = 20.75, CI 95% = 10.29–31.19) and between the chitosan and the autograft groups (*p* = 0.016, mean difference = 13.48, CI 95% = 3.03–23.93). The within subject test revealed a significant effect for time x group factors (*p* = 0.02) not for time alone (*p* = 0.16).

NRG1β is more highly expressed in the chitosan group compared with the autograft group 14 days after the repair with large effect size. NRG1β expression in the chitosan group is significantly lower than in uninjured nerves at 7 and 28 days, with large effect size, while for the autograft group it is significantly lower at all the time points analyzed with large effect size (see Table 1). Multiple comparison analysis for soluble NRG1β revealed significant differences between chitosan and uninjured nerve groups (*p* = 0.034, mean difference = −0.38, CI 95% = (−0.73)–(−0.03)) and between autograft and uninjured nerve groups (*p* = 0.0018, mean difference = −0.70, CI 95% = (−1.04)–(−0.35)). 

The within subject test revealed a significant effect for time x group factors (*p* = 0.035) not for time alone (*p* = 0.44).

### 3.4. Primary Cultures of Nerve Fibroblasts Express High Levels of NRG1 Isoforms, While NRG1 Receptors Are Not Expressed 

**Figure 3:**
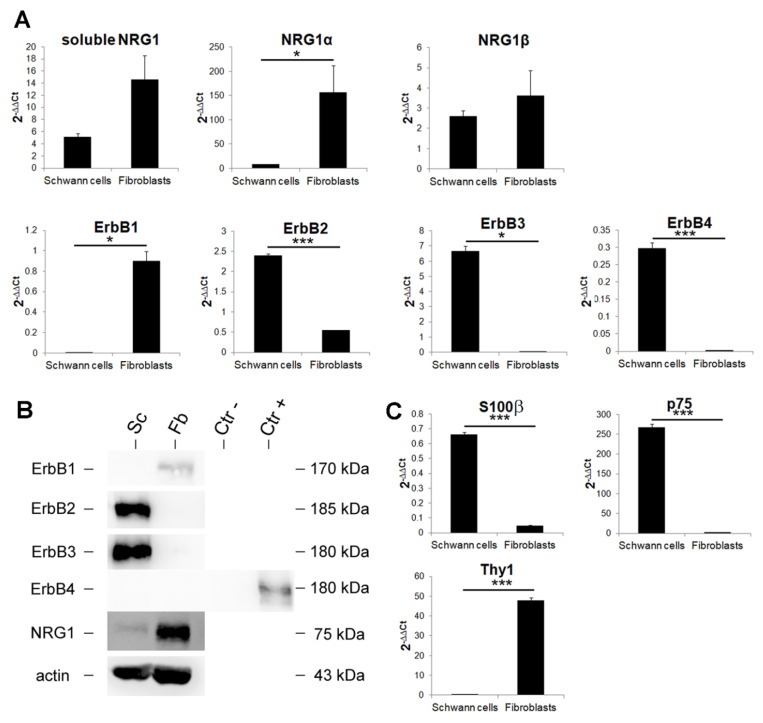
Nerve fibroblasts express high levels of soluble NRG1. (**A**) Quantitative analysis of the NRG1/ErbB system expression in primary cultures of Schwann cells and nerve fibroblasts. Relative quantification (2^−ΔΔCt^) of different NRG1 isoforms (soluble, α, β), ErbB1, ErbB2, ErbB3, and ErbB4 was evaluated by qRT-PCR. (**B**) Western blot analysis of the NRG1/ErbB system. Proteins were extracted from primary cultures of Schwann cells (Sc) and nerve fibroblasts (Fb), separated on 8% acrylamide-bis-acrylamide gels and probed with antibodies for ErbB1, ErbB2, ErbB3, and ErbB4; for NRG1, an antibody recognizing the C terminus fragment was used. Actin was used as a loading control. Size markers are indicated on the right. Neural progenitor cells stably expressing ErbB4 or mock cells were used respectively as positive (Ctr+) and negative (Ctr-) controls for ErbB4. (**C**) Quantitative expression analysis of Schwann cell and nerve fibroblast markers. Relative quantification (2^−ΔΔCt^) of S100β and p75 (Schwann cell markers) and Thy1 (fibroblast marker) was evaluated by qRT-PCR to ensure the purity of the primary cultures. For panel A and C, the geometric average of housekeeping genes ANKRD27 and RICTOR was used to normalize data. All data were calibrated to healthy median nerves, whose expression, not shown, is = 1. Values in the graphics are expressed as mean + SEM (*n* = 3 for each group). For normally distributed data with comparable variances two-tailed Student’s *t*-test was carried out, while for nonparametric data Mann–Whitney U-test was used; * *p* ≤ 0.05, *** *p* ≤ 0.001.

As the expression of Schwann cell markers was very low in the chitosan conduit, while the fibroblast marker Thy1 and NRG1 were strongly expressed, we hypothesized that fibroblasts could be involved in NRG1 expression. To test this hypothesis, confluent primary cultures of fibroblasts and Schwann cells were obtained from adult rat sciatic nerves and were analyzed at both mRNA and protein level to investigate fibroblast involvement in NRG1 expression (Figure 3A,B). 

The identity of both cell types was confirmed by qRT-PCR using specific biomarkers: S100β and p75 for Schwann cells and Thy1 for fibroblasts (Figure 3C).

At mRNA level we observed that NRG1α is more highly expressed by nerve fibroblasts in comparison with Schwann cells, while soluble and β NRG1 isoforms are highly expressed in both cell types without significant differences (Figure 3A). 

At the protein level, NRG1 is more highly expressed in nerve fibroblasts than in Schwann cells (Figure 3B); NRG1 was detected with an antibody which recognizes the 75-kDa band corresponding to the C terminal fragment of NRG1 [17,18].

Concerning ErbB receptor mRNA expression, ErbB1 is expressed by fibroblasts only, ErbB2 is more highly expressed by Schwann cells, ErbB3 and ErbB4 (NRG1 receptors) are only expressed by Schwann cells, at high and very low level respectively. 

At protein level, fibroblasts express only ErbB1, Schwann cells express only ErbB2-ErbB3, ErbB4 is not detectable in both cell types; as a positive and negative controls for ErbB4 Western blot, neural progenitor cells stably expressing ErbB4 or mock cells were used [43].

### 3.5. Immunohistochemistry Analysis Shows that Nerve Fibroblasts Express NRG1 

To assess in vivo the expression of NRG1 by nerve fibroblasts, immunohistochemistry analysis was carried out 7 days after nerve injury and repair, when at the protein level NRG1 is strongly expressed, while ErbB2 and ErbB3 are not detectable. The analysis was carried in the middle of the new tissue grown inside the conduit (the chitosan conduit was removed before fixation) and in the middle of the grafted autologous nerve.

**Figure 4:**
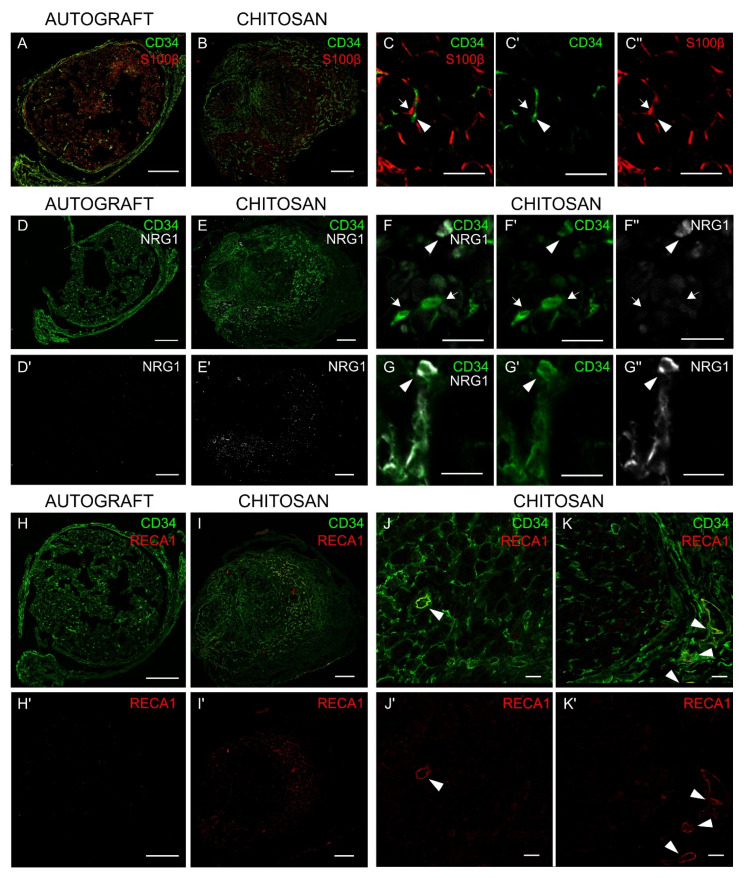
Nerve fibroblasts express NRG1 in vivo. Cross section of median nerves analyzed 7 days after repair with an autograft (**A, D, D’, H, H**’) or with a chitosan conduit (**B, E, E’, I, I’**): (**A,B**) double-stained with S100β (Schwann cell marker, red) and CD34 (fibroblast marker, green). (**C, C’, C’’**) Images at higher magnification show the absence of colocalization between S100β and CD34; fibroblasts are pointed out by arrow heads, Schwann cells by arrows. (**D,E**) double-stained with CD34 (green) and NRG1 (white) or (**D’,E’**) single-stained with NRG1; (**D’**) and (**E’**) highlight different levels of NRG1 expression in the two different experimental models (higher in the chitosan group). (**F, F’, F”** and **G, G’, G”**) Images at higher magnification show some CD34^+^ cells positive only to CD34 (arrow) and some CD34^+^ cells also positive to NRG1 (arrow head). (**H,I**) double-stained with CD34 (fibroblast and endothelial cell marker, green) and RECA1 (endothelial cell marker, red) or (**H’,I’**) single-stained with RECA1; (**H’**) and (**I’**) highlight different levels of RECA1 expression in the two different experimental models (higher in the chitosan group). (**J, J’** and **K, K’**) Images at higher magnification show some CD34^+^ cells also positive for RECA1, corresponding to endothelial cells (arrow heads), and several CD34^+^ cells positive only to CD34, corresponding to fibroblast cells. Scale bars: 200 µm (**A, B, D, D’, E, E’, H, H’, I, I’**); 20 µm (**J, J’, K, K’**); 10 µm (**C,C’,C’’, F, F’, F’’, G, G’, G’’**).

In the autograft we found several cells positive for the Schwann cell marker S100β (Figure 4A), while the signal of the fibroblast marker CD34 was very low; on the contrary, inside the conduit (Figure 4B), the signal of the Schwann cell marker was very low, while the signal of the fibroblast marker was highly detected. CD34 antibody was suitable for this analysis because it stains nerve fibroblasts [37], but not Schwann cells (as demonstrated in Figure 4, panels C-C′-C″). 

Consistent with mRNA and protein analysis, several cells were stained with NRG1 antibody in the chitosan group (Figure 4, panels E-E’), while NRG1 staining in the autograft was very low (Figure 4, panels D-D’). At higher magnification, some cells were positive for both CD34 and NRG1 (Figure 4, panels F-F’-F”-G-G’-G”). 

CD34 does not only recognize nerve fibroblast, but also endothelial cells [37]; usually, this does not represent a problem, because vessels are not very abundant in the nerve and blood vessel cross sections can be easily recognized for their rounded shape; nevertheless, because vessels in the chitosan were found to be very abundant, to unequivocally discriminate nerve fibroblasts from endothelial cells, we analyzed autograft and chitosan samples also with CD34 and RECA1 antibodies, as RECA1 antibody specifically stains endothelial cells. Data show that in the chitosan group (Figure 4, panels I-I’), unlike in the autograft (Figure 4, panels H-H’), there are several fibroblasts and several endothelial cells. At higher magnification (Figure 4, panels J-J’-K-K’) it is possible to identify both cell types: endothelial cells that are positive for both CD34 and RECA1 staining (yellow signal) and nerve fibroblasts solely positive for CD34 staining (green signal).

## 4. Discussion

For short nerve gap repair in human, up to 3 cm [8,9], tubulization is successfully used as a good alternative to autograft, especially in small diameter, noncritical sensory nerves [44], while short nerve gaps are successfully repaired in rat up to 1 cm [10,25,45]. Because several research groups [29], including ours [30], are making efforts to functionalize and enrich tubular conduits to further promote nerve regeneration in more complex situations, such as longer nerve gap or delayed repair, we focused our attention on investigating what occurs inside a hollow conduit after nerve gap repair. 

Soluble NRG1 is one of the factors known to play an important role during nerve regeneration [16]; we and others previously showed [17,18,46] that soluble NRG1 transcript is strongly and transiently up-regulated in the distal portion of the nerve 1 day after injury and that the NRG1 receptor ErbB3 is strongly down-regulated 3 days after injury and up-regulated 7, 14, and 28 days after repair, while the expression of the co-receptor ErbB2, barely detectable in the healthy nerve, is up-regulated 7, 14, and 28 days after injury [17].

Here, we analyzed the expression of the NRG1/ErbB system inside a chitosan conduit 7, 14, and 28 days after injury and repair and we compared it with the autograft. Our data show that, while the expression of ErbB2 and ErbB3 is up-regulated in the autograft 7, 14, 28 days after injury, as occurs in the distal portion of the nerve after a crush injury or after an end-to-end repair [17], in the chitosan conduit ErbB2 and ErbB3 expression starts to be detectable 14 days after nerve repair and becomes high only 2 weeks later. The absence of ErbB expression one week after injury is consistent with the fact that the conduit needs time to be colonized by repair Schwann cells [28], which in the autograft play a key role either in the Wallerian degeneration, either in nerve regeneration, while in the conduit are mainly involved in nerve regeneration. Conversely, NRG1 expression is strongly detectable in both autograft and chitosan 7 days after nerve injury and repair. Moreover, it has been shown in vitro that NRG1 stimulation of Schwann cells activates ERK, AKT, and cJun phosphorylation [47,48,49]; the lack of ERK, AKT, and cJun phosphorylation in the chitosan samples, despite the presence of high levels of NRG1, might be due to the absence (or low presence) of Schwann cells.

We further confirmed this by analysis, at mRNA level, the expression of Schwann cell markers, together with a marker of nerve fibroblasts, which are supposed to colonize tubular nerve conduits earlier, as in the first stages conduits are filled with extracellular matrix and fibrin cables [28]. The high expression of NRG1 and of a fibroblast marker together with the low expression of Schwann cell markers led us to speculate that nerve fibroblasts might be the main source of soluble NRG1 detected within the conduit. As different soluble NRG1 isoforms are known to be expressed following a nerve injury [17], we analyzed at mRNA level the relative expression of soluble NRG1 and of NRG1α and NRG1β, two soluble NRG1 isoforms characterized by different expression pattern and activity [43]. Intriguingly, we found that the expression pattern of all soluble NRG1 isoforms has a trend similar to the expression of the fibroblast marker Thy1, especially NRG1α, whose expression is strongly upregulated in the chitosan group 14 and 28 days after repair, like the fibroblast marker Thy1. The trend is similar also for NRG1β, but its expression is lower. These data are highly reminiscent of our previous observation (carried out on the distal portion of different experimental rat models: Crush injury and cut followed by end-to-end repair) where we found that NRG1α isoforms are more highly upregulated than NRG1β isoforms [17], thus reinforcing the idea that α isoforms also might play a role in nerve regeneration [50].

Protein and mRNA data do not correspond exactly: At the protein level a stronger expression of NRG1 in the chitosan samples is observed 7 days after repair, while at the mRNA level the expression is higher 14 days after repair. This discrepancy might be explained in two ways: By the fact that mRNA and protein expression could be different because of post-transcriptional and post-translational regulation or by the fact that at the mRNA level the expression of specific isoforms (soluble, α, β) is quantifiable, while no effective antibodies specific for the soluble fragment of NRG1 or for α or β isoforms are commercially available. The antibody used for Western blot analysis, acknowledged by the scientific community as a very good antibody to detect the C terminus of the transmembrane precursor of soluble NRG1 isoforms [18,40], recognizes only a subcategory of soluble NRG1 isoforms, but there are also isoforms expressed as soluble proteins without a transmembrane precursor or with a precursor with a different C terminus.

To identify the different NRG1 isoforms and NRG1 receptors and co-receptors expressed by nerve fibroblasts, an accurate mRNA and protein analysis was carried out on primary cultures of nerve fibroblasts and compared with primary cultures of Schwann cells and with healthy median nerves (used as calibrator). Our results show that nerve fibroblasts express high levels of different NRG1 isoforms: Soluble, type α and type β. Intriguingly, NRG1α isoforms are significantly more highly expressed in fibroblasts than in Schwann cells and healthy nerve, while NRG1β expression is more similar in the two primary cultures and in the healthy nerve, thus reinforcing the idea that the soluble NRG1 we observe upregulated in the chitosan might be mainly the NRG1α expressed by fibroblasts. 

Fibroblasts express ErbB1, while they do not express the NRG1 receptors ErbB3 and ErbB4 and the co-receptor ErbB2, thus suggesting that nerve fibroblasts are not able to activate a signal transduction pathway after NRG1 stimulation, which is in agreement with the lack of ERK, AKT and cJun phosphorylation. Schwann cells express only ErbB2-ErbB3 co-receptors; at mRNA level, a signal for ErbB4 is detectable, but at the protein level it is not expressed, as previously shown [51]. These results are consistent with literature data obtained from in vitro study showing that primary cultures of nerve fibroblasts can release soluble Schwann cell pro-migratory factors, including NRG1 [52,53]. However, our in vitro study demonstrated also that fibroblasts express both NRG1α and NRG1β, while Schwann cells express mainly NRG1β. Our in vivo analysis, showing high levels of the fibroblast marker Thy1 and high levels of NRG1α, suggests that nerve fibroblasts might be the main source of soluble NRG1 inside the conduit during nerve regeneration. 

To further investigate NRG1 expression in vivo, immunohistochemistry analyses were carried out 7 days after injury and repair and it was observed a high NRG1 expression in the chitosan conduit samples, while in the autograft the NRG1 signal was lower, consistently with Western blot analysis. Moreover, in the autograft samples, Schwann cells were more abundant than fibroblasts, unlike in the chitosan samples, where fibroblasts were more abundant than Schwann cells.

To verify that the high amount of NRG1 observed in the conduit might be attributed to the high presence of fibroblasts, double labelling of a fibroblast marker and NRG1 was performed. Our data show that some fibroblasts were also positive for NRG1. 

Thy1 is a good marker for mRNA analysis, but it is not suitable for IHC or Western blot analysis because it is also expressed by neurons [54]: When we analyze RNA in the distal portion of peripheral nerves (which does not include neuron cell bodies), we are analyzing RNA belonging to resident cells only (e.g., Schwann cells, fibroblasts, endothelial cells, and macrophages), while when we analyze proteins we detect proteins belonging to either the neurons, either resident cells, because neuron proteins are expressed also along the axons. 

According to Richard and colleagues [55], the sialylated transmembrane glycoprotein CD34 is a good marker of nerve fibroblasts for IHC analysis, since it is not expressed by Schwann cells, macrophages, and perineurial cells. Nevertheless, CD34 is expressed not only by fibroblasts, but also by endothelial cells, which usually are not very abundant in the nerves and can be easily recognized for the rounded shape of blood vessels and capillary. To unequivocally discriminate between fibroblasts and endothelial cells, a double staining was carried out for CD34 and RECA1, which specifically detects endothelial cells, showing that inside the conduit there are several fibroblasts, but also several endothelial cells forming blood vessels. The presence of a high number of fibroblasts and blood vessels inside the chitosan conduit is consistent with the observation that fibroblasts might promote angiogenesis by secreting cytokines [56]. Our data show that in the chitosan conduits blood vessels are highly abundant 7 days after injury and repair, while their presence is very low in the autograft samples. It has been shown by others [14] that in the bridge formed between proximal and distal stumps, when the gap is small, blood vessels provide a track for Schwann cell migration. The high presence of blood vessels in the conduit suggests that they could play here the same role. Moreover, as vascularization is also important to supply oxygen and nutrients for nerve tissue survival, different research groups are developing new strategies to further promote vessel formation inside conduits [57].

Although it has been previously demonstrated that fibroblasts accumulated at the injury site secrete extracellular matrix and form scar tissue, which might impair nerve regeneration [58], other authors demonstrated that grafted nerve fibroblasts can positively influence Schwann cell behavior during nerve repair [59]. Moreover, it has been shown that in the “nerve bridge”, formed between proximal and distal stumps after nerve injury, the interaction between nerve fibroblasts and Schwann cells—mediated by ephrinB2-EphB2—plays a key role for cell sorting and for Schwann cell cord formation [3]. 

Although hollow conduits are suitable for short gap repair, for long gaps they are unable to provide an adequate microenvironment for regeneration [60]; one possible reason might be that fibroblasts are not numerous enough to produce sufficient fibrin matrix, NRG1 and other factors. Indeed, it has been shown that matrix diameter is inversely proportional to the conduit length, deeply affecting the regenerative outcome after nerve transection [61] and that soluble NRG1 plays an important role in Schwann cell dedifferentiation, proliferation, and migration [16,18,62]. This study allows us to make two further methodological considerations: 

1-The presence of Thy1 and NRG1α also in the autograft samples, and the high levels of NRG1α (together with NRG1β) previously detected after crush injury or end-to-end repair [17] suggest that nerve fibroblasts might participate, together with Schwann cells, to NRG1 expression after nerve injury. The fact that soluble NRG1 might be produced not only by Schwann cells but also by fibroblasts, suggests that when CRE-lox technology is used to switch off NRG1 expression specifically in Schwann cells (using Schwann cell specific promoters for CRE expression), soluble NRG1 released by fibroblasts might be present in the environment and partially compensate for the absence of Schwann cell NRG1. To completely inactivate NRG1 expression (in, e.g., Schwann cells, neurons, and fibroblasts) we suggest to use inducible knockout animals, like those developed by Fricker and colleagues, ubiquitously expressing a tamoxifen inducible CRE protein which removes floxed NRG1 in all cells after tamoxifen treatment [63].

2-To obtain neuron specific gene expression or neuron specific CRE mediated gene inactivation, the neuron specific promoter Thy1 is often used, being expressed in motoneurons and DRG neurons [64]. Nevertheless, because Thy1 is expressed also by nerve fibroblasts, the use of this promoter could give rise to unspecific effects that need to be regarded, considering that fibroblasts might be involved in the regeneration process. 

## 5. Conclusions

To conclude, our results suggest that nerve fibroblasts might contribute to the high level of soluble NRG1 observed after nerve injury and repair thus promoting Schwann cell dedifferentiation. Yet, it is possible to hypothesize that nerve fibroblast grafting within conduits might be a good strategy to further promote nerve regeneration after long gap and/or delayed nerve repair.

## Figures and Tables

**Table 1:** ANOVA for repeated measures with Bonferroni’s correction.

	**Autograft vs. Chitosan**								
	**7 Days**			**14 Days**			**28 Days**		
	**η_p_^2^**	***p***	**CI 95%**	**η_p_^2^**	***p***	**CI 95%**	**η_p_^2^**	***p***	**CI 95%**
S100β	0.85	0.001	0.1–0.24	0.87	0.001	0.11–0.25	0.63	0.02	0.35–0.26
p75	0.97	0.000	64.99–93.51	0.81	0.002	24.70–71.92	0.67	0.01	4.21–23.61
Thy1	0.05	0.59	(−5.67)–9.11	0.87	0.001	(−14.38)–(−6.27)	0.87	0.001	(−15.04)–(−6.70)
sNRG1	0.00	0.907	(−1.82)–2.02	0.93	0.000	(−6.16)–(−3.71)	0.85	0.000	(−1.43)–(−0.65)
NRG1α	0.11	0.42	(−10.58)–22.33	0.69	0.01	(−51.10)–(−10.24)	0.94	0.000	(−19.71)–(−11.59)
NRG1β	0.20	0.738	(−0.46)–0.61	0.78	0.004	(−1.20)–(−0.37)	0.47	0.06	(−0.51)–0.47
	**Control vs. Autograft**								
	**7 Days**			**14 Days**			**28 Days**		
	**η_p_^2^**	***p***	**CI 95%**	**η_p_^2^**	***p***	**CI 95%**	**η_p_^2^**	***p***	**CI 95%**
S100β	0.99	0.000	0.92–1.11	0.99	0.000	0.92–1.06	0.99	0.000	0.82–1.04
p75	0.97	0.000	72.65–101.16	0.83	0.002	28.37–75.59	0.87	0.001	14.70–34.11
Thy1	0.4	0.09	(−13.5)–1.28	0.94	0.000	(−20.22)–(−12.12)	0.91	0.000	(−17.46)–(−9.12)
sNRG1	0.08	0.45	(−1.18)–2.41	0.10	0.4	(−1.59)–(−0.72)	0.50	0.033	(−0.77)–(−0.05)
NRG1α	0.48	0.06	(−0.71)–32.21	0.06	0.57	(–15.39)–25.47	0.06	0.57	(−3.07)–5.05
NRG1β	0.55	0.03	(−1.14)–(−0.06)	0.80	0.003	(−1.26)–(−0.42)	0.87	0.01	(−0.90)–( −0.41)
	**Control vs. Chitosan**								
	**7 Days**			**14 Days**			**28 Days**		
	**η_p_^2^**	***p***	**CI 95%**	**η_p_^2^**	***p***	**CI 95%**	**η_p_^2^**	***p***	**CI 95%**
S100β	0.99	0.000	(−1.05)–(−0.91)	0.99	0.000	(−1.06)–(−0.92)	0.99	0.000	(−1.04)–(−0.82)
p75	0.22	0.237	(−21.91)–6.6	0.024	0.717	(−27.28)–19.94	0.54	0.04	(−20.21)–(−0.79)
Thy1	0.4	0.09	(−1.28)–13.5	0.94	0.000	12.12–20.22	0.91	0.000	17.46–0.91
sNRG1	0.06	0.52	(−2.31)–1.28	0.92	0.000	(−5.64)–(−3.34)	0.97	0.000	(−0.95)–(−0.27)
NRG1α	0.26	0.19	(−26.34)–6.57	0.75	0.005	(−56.14)–(−15.28)	0.94	0.000	(−20.71)–(−12.57)
NRG1β	0.61	0.022	0.14–1.22	0.02	0.75	0.36–0.48	0.73	0.007	0.16–0.66

Effect of time, experimental groups, and their interaction are reported at each time point analyzed. In this table the effect size (partial eta-squared, η_p_^2^), the significance (*p*) and the 95% confidence interval (CI) are shown. The effect of the experimental groups on the regulation of gene expression is significant and with an effect size higher than expected in most conditions.

## Supplementary Materials

The following are available online at https://www.mdpi.com/2073-4409/9/6/1366/s1, Figure S1: Macroscopic view of implantation sites; rat median nerve 8 mm defect was repaired with a 10 mm long chitosan conduit (A) or with autograft technique (B). Table S1. Antibodies used for Western blot and immunohistochemistry analysis.

## Abbreviations

ANKRD27	Ankyrin repeat domain 27
ANOVA	Analysis of variance
CI	confidence interval
DAAO	D-amino acid oxidase
DMEM	Dulbecco Modified Eagle Medium
GFAP	glial fibrillary acidic protein
η_p_^2^	partial eta-squared
ERK	extracellular signal-regulated kinase
FBS	fetal bovine serum
IHC	immunohistochemistry
MAPK	mitogen-activated protein kinase
MBP	Myelin basic protein
NDS	Normal Donkey Serum
NRG1	Neuregulin1
OCT	Optimal Cutting Temperature
PBS	Phosphate Buffered Saline
PI3K	Phosphoinositide 3-Kinase
PLL	poly-L-lysine
RICTOR	RPTOR Independent Companion Of MTOR, Complex 2
SEM	standard error of the mean
VEGF	Vascular-Endothelial Growth Factor.
