# Supplementary material for: Fibroblasts Colonizing Nerve Conduits Express High Levels of Soluble Neuregulin1, a Factor Promoting Schwann Cell Dedifferentiation
Source: Cells. 2020 Jun 1;9(6):1366. doi: 10.3390/cells9061366 (PMC7349576; doi:10.3390/cells9061366)
Supplement: Supplementary file 1 [file cells-09-01366-s001.pdf]

Supplementary Information

Fibroblasts colonizing nerve conduits express high levels of soluble Neuregulin1, a factor promoting Schwann cell dedifferentiation

Benedetta Elena Fornasari[1,2], Marwa El Soury[1,2], Giulia Nato[2,3], Alessia Fucini[1], Giacomo Carta[1,2], Giulia Ronchi[1,2], Alessandro Crosio[1,2,4], Isabelle Perroteau[1], Stefano Geuna[1,2], Stefania Raimondo[1,2], Giovanna Gambarotta[1,2*]

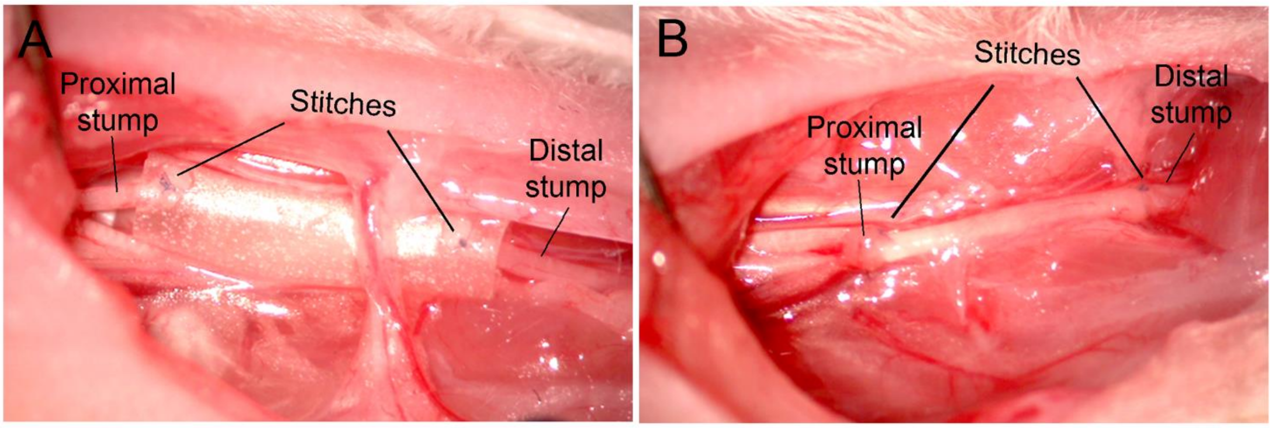

Figure 1. Macroscopic view of implantation sites. Rat median nerve 8 mm defect was repaired with a 10 mm long chitosan conduit (A) or with autograft technique (B).

Table 1. *Antibodies used for western blot and immunohistochemistry analysis* (Abcam, *Cambridge, UK;* Bio-Rad, *Hercules, CA, USA;* Cell Signaling Technology, *Danvers, MA, USA;* Covance, *BioLegend, San Diego, CA, USA;* Jackson ImmunoResearch, *Philadelphia, PA, USA;* R&D Systems, *Minneapolis, MN, USA;* Santa Cruz Biotechnology, *Dallas, TX, USA;* Sigma-Aldrich, *Merck, Darmstadt, Germany).*

Antibodies for Western blot analysis				
Primary antibodies				
	**Code**	**Dilution**	**Host**	**Source**
**AKT**	9272	1:1000	Rabbit	Cell Signaling Technology
**phospho-AKT (Ser473)**	4051	1:1000	Mouse	Cell Signaling Technology
**phospho-cJun (Ser63)**	2361	1:1000	Rabbit	Cell Signaling Technology
**HER1/ErbB1**	sc-03	1:1000	Rabbit	Santa Cruz Biotechnology
**HER2/ErbB2**	sc-284	1:1000	Rabbit	Santa Cruz Biotechnology
**HER3/ErbB3**	sc-285	1:1000	Rabbit	Santa Cruz Biotechnology
**HER4/ErbB4**	sc-283	1:1000	Rabbit	Santa Cruz Biotechnology
**ERK**	9102	1:1000	Rabbit	Cell Signaling Technology
**phospho-ERK (Thr202/Tyr204)**	9106	1:2000	Mouse	Cell Signaling Technology
**NRG1 C terminus**	sc-348	1:1000	Rabbit	Santa Cruz Biotechnology
**MBP**	SMI94 SMI99	1:4000	Mouse	Covance, BioLegend
**actin**	A5316	1:4000	Mouse	Sigma-Aldrich, Merck
Secondary antibodies				
	**Code**	**Dilution**	**Host**	**Source**

**HRP coniugated-anti-rabbit**	7074	1:15000	Goat	Cell Signaling Technology
**HRP coniugated-anti-mouse**	7076	1:15000	Goat	Cell Signaling Technology

Antibodies for Immunohistochemistry				
Primary antibodies				
	**Code**	**Dilution**	**Host**	**Source**
**CD34**	AF4117	1:100	Goat	R&D Systems
**S100β**	HPA015768	1:500	Rabbit	Sigma-Aldrich, Merck
**NRG1 C terminus**	sc-348	1:200	Rabbit	Santa Cruz Biotechnology
**RECA1**	MCA970R	1:500	Mouse	Bio-Rad (AbD Serotec)
Secondary antibodies				
	**Code**	**Dilution**	**Host**	**Source**
**AlexaFluor 488 Anti-Goat**	ab150129	1:400	Donkey	Abcam
**Cy3 Anti-Mouse**	715-165-151	1:800	Donkey	Jackson ImmunoResearch
**AlexaFluor 647 Anti-Rabbit**	711-605-152	1:800	Donkey	Jackson ImmunoResearch
